# The Ribosome as a Missing Link in Prebiotic Evolution III: Over-Representation of tRNA- and rRNA-Like Sequences and Plieofunctionality of Ribosome-Related Molecules Argues for the Evolution of Primitive Genomes from Ribosomal RNA Modules

**DOI:** 10.3390/ijms20010140

**Published:** 2019-01-02

**Authors:** Robert Root-Bernstein, Meredith Root-Bernstein

**Affiliations:** 1 Department of Physiology, Michigan State University, 567 Wilson Road, Room 2201, East Lansing, MI 48824, USA; rootbern@msu.edu; 2 UMR SAD-APT, INRA, AgroParisTech, Université Paris-Saclay, 78850 Grignon, France

**Keywords:** ribosome, rRNA, mRNA, tRNA, SINE, LINE, genome, ribosome-binding protein, aminoacyl tRNA synthetase, extra-ribosomal, non-canonical, paralog

## Abstract

We propose that ribosomal RNA (rRNA) formed the basis of the first cellular genomes, and provide evidence from a review of relevant literature and proteonomic tests. We have proposed previously that the ribosome may represent the vestige of the first self-replicating entity in which rRNAs also functioned as genes that were transcribed into functional messenger RNAs (mRNAs) encoding ribosomal proteins. rRNAs also encoded polymerases to replicate itself and a full complement of the transfer RNAs (tRNAs) required to translate its genes. We explore here a further prediction of our “ribosome-first” theory: the ribosomal genome provided the basis for the first cellular genomes. Modern genomes should therefore contain an unexpectedly large percentage of tRNA- and rRNA-like modules derived from both sense and antisense reading frames, and these should encode non-ribosomal proteins, as well as ribosomal ones with key cell functions. Ribosomal proteins should also have been co-opted by cellular evolution to play extra-ribosomal functions. We review existing literature supporting these predictions. We provide additional, new data demonstrating that rRNA-like sequences occur at significantly higher frequencies than predicted on the basis of mRNA duplications or randomized RNA sequences. These data support our “ribosome-first” theory of cellular evolution.

## 1. Introduction: The “Ribosome-First” Theory Predicts that tRNA- and rRNA-Like Genes should be Over-Represented in Cellular Genomes

Orthologous genes conserved across all species constitute what is called the Universal Gene Set of Life (UGSL), and consists of less than 100 genes [1,2,3]. Not surprisingly, the UGSL is dominated by translation-related genes. These ribosomal RNAs (rRNAs), transfer RNAs (tRNAs), and ribosomal proteins make up the most abundant macromolecular species in all living organisms [4,5]. More surprisingly, as we will demonstrate here, macromolecules based on rRNA-like, tRNA-like and ribosomal-protein-like modules also dominate the rest of the genome, playing roles far removed from translation. We argue that this over-abundance of ribosome-like sequences can only be explained if ribosomes evolved prior to cells providing, through an rRNA-based genome, the basis from which subsequent cellular genomes were elaborated. We call this ribosomal origin of the genome the “ribosome-first” theory of the origins of cellular life.

This “ribosome first” theory of the origin of cellular genomes follows directly from our previous work, in which we have suggested that ribosomes were the first self-replicating entities from which modern cells evolved. The “ribosome-first” hypothesis makes a series of surprising predictions, including the necessity for rRNA to encode genetic information containing ribosomal-protein-related functions; to function as messenger RNA (mRNA) as well as rRNA; and to encode the tRNAs that are required to implement translation. In previous papers, we demonstrated that rRNA does appear to encode, in one or more reading frames, the genetic information required to produce many of the active-site protein segments that are associated with its own functions (including ribosomal proteins, polymerases, ligases, amino acid transferases, and phosphatases). The rRNA also encodes at least two complete sets of tRNAs to translate its own sequence into these protein segments. Also, rRNA appears to have the properties of messenger RNAs (mRNAs). In some cases, rRNA has retained this mRNA function by encoding proteins that are translated in modern organisms. In addition, modern ribosome-binding proteins, as well as many amino acid transferases (synthetases) have the unique property of autologously regulating their own synthesis by binding to their own mRNA. We attribute this autologous control feature to the evolution of a self-sufficient regulatory network of molecular interactions within the ribosomal self-replication schema [6,7,8]. These observations alter the basic conception of the ribosome from a specialized molecular machine that evolved, following an established RNA-world to one in which the ribosome is a vestigial remnant of a prebiotic self-replicating entity that contained genes, maintained a limited metabolism, and evolved into the first cells.

A logical prediction of our “ribosome-first” theory of the origins of cellular life, which we explore for the first time here, is that the ribosomal genome preceded the evolution of the cellular genome and gave rise to it. Divergent evolution of the ribosomal genes through duplication and modification would have produced the fundamental core of modern cellular genomes. Ribosomal RNAs themselves appear to have evolved from the ligation of multiple tRNA-like modules [6,9,10,11] Modern genomes should therefore contain an unexpectedly large percentage of tRNA- and rRNA-like modules derived from both sense and antisense reading frames. These molecules should therefore have been adapted to play multiple roles in primitive cells and should therefore be plieofunctional. Moreover, divergent evolution should have dispersed these tRNA- and rRNA-like genetic elements so that they should be found to be embedded within non-ribosomal proteins with key functions within the cell. In this paper, we review existing literature that provides evidence for all of these predictions of the ribosome-first hypothesis, and provide a series of proteonomic tests of this hypothesis that distinguish its predictions from those derived from other possible hypotheses.

Testing the ribosome-first theory of genome evolution (or any other theory) requires not only marshalling evidence for it, but equally importantly, distinguishing between the predictions made from the theory being tested, and the predictions that stem from other possible explanations of cellular evolution. In general, origins of life theories fall into three categories: (1) RNA- or more broadly, “genome-first” theories; (2) protein- or “metabolism-first” theories; and (3) co-evolution theories, in which genome, proteins and metabolism emerge synergistically. The “ribosome-first” theory of cellular evolution is one version of possible co-evolution theories, since it posits an RNA genome encoding the proteins necessary to make a functional ribosome that could carry out replication, transcription, and translation [6,12]

The critical differences between these general theories are as follows. In a “genome-first” theory, the ribosome evolves as a late addition to a pre-existing, functional genome that evolved from an RNA- or RNA–DNA-world. The pre-existing genome should be the basis for the emerging cellular genome, from which protein metabolism would emerge, with ribosome-related genes representing a small, specialized, late supplement to that genome. Extra-ribosomal functions for rRNAs, tRNAs, and ribosomal proteins should have evolved only rarely, and rRNAs should not contain any genetic information of their own. In this case, randomly evolved sequences should have been the basis for the first genomes with selection for functionality following from selection on the diversity of sequences.

In the “metabolism-first” theory, the cell, presumably including its diverse protein components, evolves prior to the genome or the ribosome. The genome then evolves to encode these pre-adapted proteins, such as RNA and/or DNA genes. The ribosome would, in a metabolism-first scenario, play a subsidiary role as a mere translation mechanism, and therefore, once again, be encoded by a small number of late-evolving genes, and rRNA should not contain any genetic information of their own. rRNA and ribosomal protein-related sequences should, once again, be rarely encountered in the wider genome and ribosome-related proteins should only rarely have evolved extra-ribosomal functions. Instead, protein-encoding genes should be found to be over-represented and modified throughout the genome.

However, in a “ribosome-first” theory, the first self-replicating organism is the ribosome itself, and the first genome that of the ribosome. Thus, both protein metabolism and a core genome pre-date the evolution of cells. In the case of the ribosome-first theory, this integration specifically arises within the ribosome complex itself, such that rRNA-encoded genes and ribosome-related proteins and their metabolic functions emerge as a tightly knit network or interactome. This interactome should be the basis from which many cellular functions evolve, and therefore the cellular genome would have evolved from this small set of ribosome-related genes. Moreover, rRNAs, tRNAs, and ribosomal proteins would have been co-opted to carry out key non-translation related functions during cellular evolution. In short, the “ribosome-first” theory predicts that ribosome-like genes will be significantly over-represented in cellular genomes, and that rRNA, tRNA and ribosomal proteins should all be plieofunctional.

The purpose of this article is to aggregate and review various types of evidence acquired by many investigations of the nature of genome organization in light of the genes-first, metabolism-first and ribosome-first theories, and to provide proteonomic tests of whether ribosome-related genes are significantly over-represented in cellular genomes, as compared with what one might expect from random evolution of sequences or from a protein-first origin.

## 2. Results

### 2.1. Eukaryotic and Prokaryotic Genomes Contain Unexpectedly Large Numbers of rRNA-Like Sequences

All cellular genomes contain unexpectedly large numbers of rRNA-like sequences, an observation that was first reported by Mauro and Edelman in 1997 for eukaryotes [13]: “Many eukaryotic mRNAs contain sequences that resemble segments of 28S and 18S rRNAs, and these rRNA-like sequences are present in both the sense and antisense orientations. Some are similar to highly conserved regions of the rRNAs, whereas others have sequence similarities to expansion segments. In particular, four 18S rRNA-like sequences are found in several hundred different genes, and the location of these four sequences within the various genes is not random. One of these rRNA-like sequences is preferentially located within protein coding regions immediately upstream of the termination codon of a number of genes…. We consider the hypotheses that rRNA-like sequences may have spread throughout eukaryotic genomes and that their presence in primary transcripts may differentially affect gene expression.”

Several lines of evidence supported Mauro and Edelman’s conjecture. Mauro and Edelman themselves demonstrated that regions within mRNAs that were complementary to rRNA bound to rRNAs in the ribosome resulting in translational control of gene products. The Gtx homeodomain protein is one well-characterized example [14,15,16]. Several investigators have also demonstrated the presence of rRNA-like sequences in a wide array of poly-adenylated mRNAs. Poly-adenylation is a process in which adenines are added to the end of mRNA during transcription, to aid in the transport of the mRNA to the ribosome, and to identify the RNA as carrying a translatable message. Thus, the presence of rRNA-like sequences in polyadenylated RNAs provides further evidence of a role for rRNA sequences in identifying functional proteins.

Mauro and Edelman, for example, found that [13]: “Northern blot analysis of poly(A) RNA from different vertebrates (chicken, cattle, rat, mouse, and human) revealed that a large number of discrete RNA molecules hybridize at high stringency to cloned probes prepared from the 28S or 18S rRNA sequences that were found to match those in mRNAs. Inhibition of polymerase II activity, which prevents the synthesis of most mRNAs, abolished most of the hybridization to the rRNA probes.” Kong, et al. [17] verified that rRNA-like sequences are unusually abundant in poly-adenylated mRNA. Kong, et al. [17] also identified two different types of such poly-adenylated rRNA-containing transcripts (PART). Type I PART contain sequences of tens to several hundreds of rRNA-associated nucleotides within the transcript itself, while Type II PART contain very long, sometimes almost complete, rRNA sequences with a 5′ cap and a 3′ polyadenylation sequence. PART I include tRNA synthetases, RNA-binding proteins, insulin-like growth factor binding proteins, tumor necrosis factor, homeobox genes, heat shock 86 protein, connexins, glycine methyltransferase, nuclear respiratory factor 1 (NRF 1), and many other classes of proteins that are essential to cell functions. PART II include mitochondrial leucyl-tRNA synthetase, which is 98% identical to a 396 nucleotide stretch of the 28S rRNA; phosphocan short isoform (RPTP-beta gene), which is 98% of 1537 nucleotides of the 28S rRNA; superoxide dismutase 1 (SOD1) upregulated mRNA 1, which is identical to the first 1700 of 1879 nucleotides of 18S rRNA; human humanin (HN1), which is identical to 1553 nucleotides of the 28S rRNA (see also [18]); human serine/threonine protein kinase Kp78, CTAK75a, which is 99% identical to 905 nucleotides of the 18S rRNA; mouse ETS-related transcription factor ERF (Erf1), which is 95% identical to a 705 nucleotide stretch of the 18S rRNA; mouse phosphacan short isoform (RPTP-beta gene), which is 98% identical to 1573 nucleotides of the 28S rRNA; human succinate dehydrogenase complex, subunit A, which is 99% identical to 643 nucleotides of the 18S rRNA [17]. Kong, et al., conclude that, “It appears that rRNA segments are dispersed throughout the genome” [17].

Additional proteins that share significant portions of rRNA sequences have been identified by other investigators. One is the rDNA-encoded mitochondrial protein Tar1p (transcript antisense to ribosomal RNA) in *Saccharomyces cerevisiae* [19,20], which regulates glucose-related respiration. Coelho et al. argue that the existence of Tar1p may demonstrate, “that rDNA transcription and mitochondrial function are coordinately regulated in eukaryotic cells” [19]. Lee, et al. [18] add that Tar1p is likely to be only one of many such mitochondria-derived rRNA transcripts: “Many of the mRNA species identified from the mitochondria are discrete smaller length ones that do not map to the traditional mitochondrial protein-encoding genes. In particular, multiple such mRNAs are observed from the 16S rRNA region, including the site of the humanin ORF…. Using parallel analysis of RNA ends (PARE) to map the transcript cleavage sites, a plethora of expected and unexpected cleavage sites have been discovered for the mitochondria [21]. The majority of tRNAs and mRNAs have distinct dominant cleavage sites at the 5′ termini, but intragenic cleavage sites are especially abundant in rRNAs. The latter stages of precursor modification include 3′ polyadenylation of mRNAs, with the exception of the mitochondrial ND6 gene, and rRNAs, in agreement with previous reports [21,22]. In addition, mitochondrial rRNAs are transported and found in the cytoplasm with significant biological roles in, for instance, *Drosophila* germ cell establishment [23,24], and 16S rRNA was found to be localized in the nucleus of human spermatogenic cells [25]. It is possible that the polyadenylated humanin transcript is exported to the cytoplasm where it can be translated.”

Mignone and Pesole [26] have also reported very large numbers of rRNA-like sequences in the human genome, but in contradistinction to the papers just cited, have argued that the vast majority (especially of the longer, type II PART) are artifacts of homology searching, and that only a small subset of short, type I PART stand up to critical analysis of the data. Their own data analysis, however, argues against their conclusions, at least from a ribosome-first perspective. They note that while “the distribution of low-similarity matches was similar for both real and simulated rRNA sequences,… interestingly,… the long/highly similar matches can be found only with the actual rRNA sequences” ([26] p. 150). Oddly, then, they discarded all of the long sequence (type II PART) homologies as artifacts on the basis that, “The great majority of rRNA-like tracts found in human mRNAs were not supported by counterparts in genomic or EST [expressed sequence tag] sequences” ([26] p. 151)—a statement in direct contradiction to the results of Mauro and Edelman [13] and Kong [17]. In any event, Mignone’s and Pesole’s requirement that rRNA-like sequences actually be transcribed is not required by the ribosome-first theory, which demands only that rRNA-like sequences be found in unexpectedly high numbers compared to the numbers of genes that are required to encode ribosome production. It is quite possible that organismal genomes evolved from duplicated rRNAs and subsequently transferred protein-encoding functions to more specialized exon regions. We believe that this is, in fact, is exactly what Mignone and Pesole found. Additionally, our own analysis of the occurrence of rRNA-like sequences in the genome, which we provide below, reveals a possible flaw in the method by which Mignone and Pesole analyzed their results.

A third set of evidence also argues for the presence of rRNA-derived sequences in cellular genomes at unusually high rates. Gene sequences that are complementary to rRNA genes also populate the genome in unexpectedly large numbers, perhaps representing the incorporation of transcription products of rRNA “genes” into the genome. Examples include complementarity between ferritin H mRNA and 28S ribosomal RNA [27] between the avian myeloblastosis oncogene and eukaryotic 28S ribosomal RNA [28], and murine 18S rRNA with a wide range of mRNAs [29]. Such complementary sequences are not predicted by the genome-first or metabolism-first theories of cellular evolution, but they follow directly from the ribosome-first theory in which all six reading frames of rRNA are utilized to encode protein sequences.

Other data also take on different interpretations, depending on the theoretical framework from which they are interpreted. Genome-wide sequencing studies have repeatedly run into the problem of “discovering” new genes and new gene families within or across overlapping genome regions that encode rRNA. The ribosome-first theory of cellular evolution explicitly proposes that such overlaps not only should, but they must exist, since the rRNAs must contain the genes encoding for their own ribosomal proteins. Thus, the protein-encoding regions must be contained within, or overlap, the rRNA sequences [6,7,8]. No such prediction follows from the genome-first or the metabolism-first theories of cellular evolution. In a genome-first theory, the ribosome would have evolved after the core genome in order to make possible protein production, in which case protein-encoding regions that overlapped with rRNAs would interfere with pre-existing gene function. Similarly, if the ribosome evolved to optimize pre-existing protein metabolism, then key protein sequences should be the basis for rRNA evolution rather than rRNAs serving as the basis for protein evolution.

In fact, a robust literature clearly demonstrates that rRNAs do overlap both known and possible protein-encoding sequences, and there is no evidence that specific protein-encoding sequences are the origins of rRNAs. For example, metagenomic studies of RNA expression generally yield a predominance of rRNA-like sequences that can approach 90% of the total RNA sequenced [30,31,32,33,34,35]. Tripp, et al. [36] have provided a good summary of the type of data that are treated as artefactual: “In the course of analyzing 9,522,746 pyrosequencing reads from 23 stations in the Southwestern Pacific and equatorial Atlantic oceans, it came to our attention that misannotations of rRNA as proteins is now so widespread that the rate of false-positive matching of rRNA pyrosequencing reads to the National Center for Biotechnology Information (NCBI) non-redundant protein database approaches 90%. One conserved portion of 23S rRNA was consistently misannotated often enough to prompt curators at Pfam to create a spurious protein family” ([36], p. 8792). This “problem” of pseudo-proteins being encoded within rRNA sequences, or overlapping them, is so widespread that programs have been written to aid researchers in eliminating them during genome-wide sequencing experiments (e.g., [30,31,32,33,34,35]). Without challenging whether proteins are actively encoded in rRNA-encoding sequences in prokaryotes and archaea, the ribosome-first theory does force us to consider the possibility that such apparent encodings are fossils of the ribosomal origins of the genome. At the same time, the widespread existence of such inclusions and overlaps makes no sense from other genome origins theories. From an evolutionary perspective, we must confront the question of why so many “spurious” rRNA-like sequences exist.

It is also important to point out that the desire to eliminate “false” protein families from genome-wide sequencing studies is based on three key points. One is the assumption that rRNA-like sequences should not appear ubiquitously in the genome. The ribosome-first theory undercuts that assumption. The second point is that the argument for eliminating rRNA-like sequences is based on a lack of evidence that they are proper annotations. Lack of evidence is not evidence against their possible validity. Yet, this is precisely the current reasoning. As Tripp, et al. point out, “rRNA operons in Bacteria and Archaea are not known to contain naturally expressed protein coding regions that also code for rRNA” ([36] p. 8792). Tripp, et al., therefore consider that, “Annotations of Bacteria and Archaea proteins embedded in rRNA operons and overlapping with rRNA coding regions within those operons have been *rightly* [emphasis added] presumed to be misannotations [37] and should continue to be, until hard evidence to the contrary emerges” ([36] p. 8792). From a ribosome-first perspective, we would strongly urge a search for such “hard evidence”, since it seems unlikely that dual-use, overlapping protein-rRNA sequences evolved ubiquitously without some function, either past or present. Moreover, there is no rationale other than a ribosome-first origin, for the genome to explain why rRNA-like sequences would be both ubiquitous and meaningless, and that rationale would hold only if functions originally associated with rRNA genes were off-loaded onto other genes during evolution.

In fact, many dual-use genes encoding both proteins and rRNAs are known in eukaryotes [13,20,24,37,38,39]. Recently, Mumtaz and Couso [40] have used Ribo-Seq techniques to examine ribosome-bound mRNAs, and they discovered large numbers of rRNA-like sequences bound to the ribosomes that were apparently translated, and which had previously been ignored as “non-coding” sequences: “Earlier bioinformatic approaches had shown the presence of hundreds of thousands of putative small ORFs (smORFs) in eukaryotic genomes, but they had been largely ignored, due to their large numbers and the difficulty in determining their translation and function. Ribo-Seq has revealed that hundreds of putative smORFs within previously assumed long non-coding RNAs (lncRNAs) and UTRs of canonical mRNAs are associated with ribosomes, appearing to be translated” [40].

One must consider an additional hypothesis, which is that dual-use sequences may have been very common during the emergence of cellular life, but that selection pressures have largely separated rRNA-encoding sequences from protein-encoding sequences, so as to avoid regulatory confusion. The evolutionary problem that must be faced, regardless of which perspective is taken then, is to explain the origins of ubiquitous rRNA-protein overlaps, whether they are non-functional fossils or functional overlaps. This problem currently appears to be intractable from anything other than the ribosome-first theory. Indeed, this evolutionary problem exists for the origins of all rRNA-like genes within genomes. To quote Kong, et al. again: “For type II PART, there is a possibility that some of these RNA may derive from rRNA genes or rRNA. The origins of these types of RNA remain to be elucidated” [17].

### 2.2. Genomes Contain Unexpectedly Large Numbers of rRNA- and tRNA-Derived Transposon Sequences

According to the ribosome-first theory, not only should rRNA-like sequences be unusually prevalent in all cellular genomes, so should tRNA-like sequences. This prediction follows from the fact that tRNA-like sequences appear in multiple reading frames within rRNAs [6,7,8,9,10,11]. Indeed, recent research has revealed that prokaryotic and eukaryotic genomes contain large numbers of short interspersed repetitive elements (SINE) derived from fragments of rRNAs and tRNAs (SINE1) or 7SL (Alu) (SINE 2), as well as mammalian-wide interspersed repeats (MIRs). These sequences may provide evidence for how rRNA (or tRNA)-derived ribosome-encoded genes multiplied to form cellular genomes.

Alu (named after the *Arthrobacter luteus* restriction endonuclease), or SINE2, are abundant transposable elements within genomes. While the genes encoding ribosomal RNA, tRNAs, and ribosomal proteins (rDNA) comprise only 0.01% of the human genome, and exons comprise only 1.5% of the human genome [41], tRNA-like Alu and SINES make up about 11% to 15% of mammalian genomes, and are largely located in intron regions [41,42]. MIRs make up another 1–2% of most organismal genomes [43].

Investigators have also found increasing numbers of new types of SINE derived from 5S rRNA (SINE3) and 28S rRNA (SINE 28). Longo, et al. [44] provide a good summary of the new findings: “Complex eukaryotic genomes are riddled with repeated sequences whose derivation does not coincide with phylogenetic history, and thus is often unknown. Among such sequences, the capacity for transcriptional activity, coupled with the adaptive use of reverse transcription, can lead to a diverse group of genomic elements across taxa, otherwise known as selfish elements or mobile elements. Short interspersed nuclear elements (SINEs) are nonautonomous mobile elements found in eukaryotic genomes, typically derived from cellular RNAs such as tRNAs, 7SL, or 5S rRNA. Here, we identify and characterize a previously unknown SINE derived from the 3′-end of the large ribosomal subunit (LSU or 28S rDNA) and transcribed via RNA polymerase III. This new element, SINE28, is represented in low-copy numbers in the human reference genome assembly, wherein we have identified 27 discrete loci” [44]. Additional sequences derived from the 28S ribosome can be found scattered throughout the human genome in varying copy numbers [45,46].

Highly conserved 5S rRNA-derived SINEs (SINE3) have been reported in human and other mammals that Nishihara, et al. [47] have called AmnSINE1. “AmnSINE1 has a chimeric structure of a 5S rRNA and a tRNA-derived SINE, and is related to five tRNA-derived SINE families that we characterized here in the coelacanth, dogfish shark, hagfish, and amphioxus genomes. All of the newly described SINE families have a common central domain that is also shared by zebrafish SINE3, and we collectively name them the DeuSINE (Deuterostomia SINE) superfamily. Notably, of the approximately 1000 still identifiable copies of AmnSINE1 in the human genome, 105 correspond to loci phylogenetically highly conserved among mammalian orthologs.” This high degree of conservation strongly suggests evolutionary selection for functionality. Other examples of SINE3 have been found in fruit bats [48], fungi [49], and zebrafish [50] among other organisms [51]. In zebrafish, Kapitonov and Jurka report that, “SINE3s are transcribed from the type 1 internal pol III promoter. Approximately 10,000 copies of SINE3 elements are present in the zebrafish genome; they constitute approximately 0.4% of the genomic DNA” [50]. The 1000 or so SINE3 found in the human genome constitute an equivalent percentage.

In light of the fact that SINES and MIRs make up 15% to 20% of many genomes, Frenkel, et al. [52] and Schmitz [42] have proposed that SINEs may be the drivers of genome evolution. If that hypothesis is correct, then the further possibility must be entertained that the tRNA and rRNA origins of SINEs and MIRs are molecular “fossils” of the mechanism by which the original cellular genomes were formed through self-replicating, or insertion-amplifying rRNA and tRNA sequences. Such a mechanism would be entirely consistent with a ribosome-first origin of cellular genomes, but once again, this is difficult to explain from other theoretical perspectives.

### 2.3. tRNA and rRNA Control of Non-Ribosomal Cellular Functions

A further unique, testable implication of the ribosome-first theory of genome origins is that ribosomal proteins, rRNAs and tRNAs should be pleiofunctional, playing roles in cellular functions beyond translation. This prediction follows from the assumption that the ribosomal molecules were the foundation from which other core cellular molecules and their functions evolved. The interactivity of the ribosomal complex itself would have assured a high degree of interactivity among the genes and proteins that evolved from it, creating a stable interactome. Therefore ribosomally-derived molecules would have been under significant selection pressure to be retained in primordial cells, and to become adapted to the broader range of functions that the cell required. Evidence for such a scenario is abundant.

Wegrzyn and Wegrzyn [53] and Katz, et al. [54] have reviewed studies demonstrating that tRNA has multiple cellular functions beyond is canonical role in protein translation (Table 1). Various retroplasmids and retroviruses encode a reverse transcriptase that can use a tRNA sequence as the basis for synthesizing a cDNA copies. DNA replication itself is regulated by the accumulation of uncharged tRNAs that bind to, and inactivate, several repressors of DNA synthesis. The proportion of charged to uncharged tRNAs functions as a critical sensor of amino acid deficiency in both prokaryotes and eukaryotes (though through different mechanisms), mediating transcription and translation to improve the metabolic efficiency of amino acid synthesis. Also, uncharged tRNAs can act as ribozymes for the self-deletion of introns, again fostering increased metabolic efficiency under stress conditions. In addition, tRNA transcript fragments averaging about 30 to 35 nucleotides in length have been demonstrated to play diverse cellular roles, mainly in controlling transcription and translation in both prokaryotes and eukaryotes [55]. These tRNA fragments are widespread and numerous in prokaryotic [56] (and eukaryotic cells [57] under stress (see also [58,59,60,61,62]). tRNA fragments not only interact with other short RNA to regulate their activity, but also sequester RNA-binding proteins, buffering the translation system. Some tRNA-like repeats encode proteins that function specifically as Rho-dependent transcriptional regulators [55]. Finally, eukaryotic tRNA genes sometimes function as “chromatin insulators”, separating active chromatin domains from silenced ones [58]. Thus, tRNA and its fragments play important metabolic roles in replication, transcription and translation beyond simply recognizing specific codons on mRNAs and providing amino acids for ligation into proteins.

tRNA-derived small RNA (tsRNA) or tRNA fragments (tRF) have also been identified as novel regulatory RNAs controlling a wide range of physiological and pathological processes in organisms from Archaea through plants and animals [63,64,65,66]. tsRNA and tRF functions that have been identified include transcription regulation, cell proliferation, tumor genesis, stress response, intergenerational epigenetic inheritance, mRNA binding to ribosomes, amino acylation of tRNAs by amino acyl transferases, ribosomal protein function, and protection against retrotransposons such as SINEs [67,68,69,70].

Finally, tRNA participate in non-canonical forms of peptide synthesis independent of the ribosome, in conjunction with non-ribosomal peptide synthases. To quote Giessen and Marahiel, tRNA, “are also used as substrates and amino acid donors in a variety of other important cellular processes, ranging from bacterial cell wall biosynthesis, and lipid modification to protein turnover and secondary metabolite assembly” [71]. For example, tRNA^Gly^ [72] is involved in peptidoglycan synthesis, while tRNA^Lys^ participates in remodeling lipids [73]. Ribosome-free production of cyclic dipeptides (diketopiperazines), for example, results in molecules that are essential for iron chelation or other types of siderophore activity; quorum sensing in bacteria; anti-viral, anti-bacterial, or anti-fungal activity, thereby protecting organisms from their pathogens [74,75].

Notably, tRNA may also have provided the material from which rRNA evolved. 5S rRNA has significant homologies with tRNA, and probably evolved directly from one or more tRNA precursors [9,10,11,76]. Like tRNAs, the 5S rRNA has also evolved to take on broader cellular functions. The 5S rRNA has not only undergone an alternative splicing event that has exonized it within the gene of its own transcription regulator, TFIIIA (transcription factor for polymerase III A) in all land plants [77,78], but has also mutated into an RNA mimic that regulates the alternative splicing of TFIIIA [79]. The 5S rRNA can also exert extra-ribosomal function by binding to ribosomal proteins prior to their integration into functional ribosome complexes. One classic example is the control of the oncogene p53 by a 5S rRNA–RPL5–RPL11 complex that inhibits the Hdm2 protein that normally blocks p53 activity [80,81].

Like tRNA fragments, rRNA fragments (rRF) have also been found to exert a range of extra-ribosomal functions (Table 2). For example, Arkov, et al. [82] and Chernyaeva, et al. [83], “identified a fragment of the *Escherichia coli* 23S rRNA (nucleotides 74 to 136) whose expression caused readthrough of UGA nonsense mutations (but not UAA or UAG) in certain codon contexts in vivo. The antisense RNA fragment produced a similar effect…. Since termination at UGA in *E. coli* specifically requires release factor 2 (RF2), our data suggest that the fragments interfere with RF2-dependent termination” [83]. Similarly, Feagen, et al. [84] reported high levels of rRNA fragments generated as functionally active transcripts from rDNA in *Plasmodium*. Chen, et al., [85] report that, “Two series of rRFs (rRF5 and rRF3) were precisely aligned to the 5′ and 3′ ends of the 5.8S and 28S rRNA genes. The rRF5 and rRF3 series were significantly more highly expressed than the rRFs located in the body of the rRNA genes. These series contained perfectly aligned reads, the lengths of which varied progressively, with 1-bp differences. The rRF5 and rRF3 series in the same expression pattern exist ubiquitously from ticks to human. The cellular experiments showed the RNAi knockdown of one 20-nt rRF3 induced the cell apoptosis and inhibited the cell proliferation.” Shi, et al. [86] have developed a program called “SPORTS1.0”, for annotating and profiling additional non-coding RNAs optimized for rRNA- and tRNA-derived small RNAs.

rRNA fragments can also function as interference RNAs. Hadjiargyrou and Delihas [87] have found that a large proportion of non-coding RNAs with regulatory functions derive from SINE elements, which are themselves often derived from tRNA-like sequences. Thus, tRNA and rRNA fragments appear to have fundamental regulatory roles in cellular metabolism that predate the origins of cellular life and have been incorporated as essential components of cellular life. Cerutti and Casas-Mollano [88] suggest that interference functions for RNA were already in place prior to the origins of cellular life, making rRF and tRNA-derived small RNA fundamental aspects of ribosome function and evolution: “We have examined the taxonomic distribution and the phylogenetic relationship of key components of the RNA interference (RNAi) machinery in members of five eukaryotic supergroups. On the basis of the parsimony principle, our analyses suggest that a relatively complex RNAi machinery was already present in the last common ancestor of eukaryotes and consisted, at a minimum, of one Argonaute-like polypeptide, one Piwi-like protein, one Dicer, and one RNA-dependent RNA polymerase. As proposed before, the ancestral (but non-essential) role of these components may have been in defense responses against genomic parasites such as transposable elements and viruses. From a mechanistic perspective, the RNAi machinery in the eukaryotic ancestor may have been capable of both small-RNA-guided transcript degradation as well as transcriptional repression, most likely through histone modifications.”

### 2.4. Non-Ribosomal Functions of Ribosomal Proteins

As surprising as it may be that tRNAs and rRNAs and their fragments participate directly in cellular functions beyond their canonical roles, one of the strongest lines of evidence that tRNA and rRNA provide the basis for cellular genomes comes from studies of widespread non-ribosomal function for ribosomal proteins and genes. As can be seen in the next four Tables, almost all ribosomal proteins exhibit extra-ribosomal functions. We have previously reviewed evidence for autologous regulation of most ribosomal proteins of their own translation by means of binding directly to their own mRNA [7], and they include only a summary of that information in Table 3, Table 4, Table 5 and Table 6. The majority of ribosomal proteins, both in prokaryotes and eukaryotes, play additional roles in cellular metabolism, ranging from DNA repair, control of transcription and replication, and histone binding to the regulation of various enzymes and mRNA splicing as summarized in Table 3, Table 4, Table 5 and Table 6 [81,89,90,91,92,93,94,95,96,97,98,99,100,101,102,103,104,105,106,107,108,109,110,111,112]. The degree to which these extra-ribosomal functions are integrated into cellular functions is exemplified by the participation of riboprotein L13a into the GAIT complex in eukaryotes. The GAIT complex is produced in response to interferon gamma activation and consists of five components: riboprotein L13A binding to a conserved region on mRNA encoding ceruloplasmin or related inflammation response proteins, along with glutamyl-prolyl-tRNA synthetase, NS1-associated protein-1, and glyceraldehyde 3-phosphate dehydrogenase. GAIT prevents the 43S ribosome complex from integrating into a completed ribosome [81,89]. We will discuss further roles for tRNA synthetases in the next section, only noting here that it is important to realize that the artificial division that we have adopted here of summarizing extra-ribosomal function by the class of the molecule hides important interactions among these classes that are of equal importance to understanding the self-replicating ribosome as a functional interactome.

As extensive as the currently documented extra-ribosomal functions of ribosomal proteins are, new functions continue to be discovered, which has led Warner and Mcintosh to ask whether the fact that we have not found even more such functions is, “due to a lack of imaginative evolution by cells and viruses, or to a lack of imaginative experiments by molecular biologists?” [90]. Only further research can determine the answer. We predict that there are many more extra-translational ribosomal-protein functions to be found.

### 2.5. Non-Canonical Functions of Aminoacyl tRNA Synthetases (aaRS)

As noted in the previous section, yet another class of ribosome-related proteins that have important additional cellular functions are the aminoacyl tRNA synthetases. The function usually ascribed to these proteins is to conjugate the appropriate amino acid to the correct tRNA. We have previously demonstrated that key functional elements of tRNA synthetases are encoded in all six reading frames of rRNAs, so that ribosomes may have had the ability to produce both tRNAs and the core enzyme structures that are required to load them with the correct amino acids [6,8]. Caprara, et al. [113] and Mohr, et al. [114] reported that the *Neurospora crassa* mitochondrial (mt) tyrosyl-tRNA synthetase (CYT-18 protein) functions in splicing group I introns, in addition to aminoacylating tRNA(Tyr). Leucyl-tRNA synthetase has a similar function [115,116]. Lysyl-tRNA synthetase catalyzes the synthesis of dinucleoside polyphosphates in *E. coli* [117,118] while the threonyl-tRNA synthetase from the same organism exerts autogenous control on its own mRNA, to self-regulate its translation [119]. Other documented functions include the regulation of transcription, translation, splicing, inflammation (sometimes by mimicking cytokine activity) [120,121,122], angiogenesis and apoptosis [123], and cell signaling [124,125]. Some aaRSs interact specifically with other proteins [126]. For example, many of these functions are mediated in higher eukaryotes by an aaRS complex involving eight different aaRS and three aminoacyl-tRNA synthetase-interacting multifunctional proteins (AIMS). The complex is formed of ArgRS, GlnRS, IleRS, LeuRS, LysRS, MetRS, AspRS, and Glu-ProRS around a core of AIMP1, AIMP2, and AIMP3, also known as p43, p38, and p18. The release of individual proteins from the complex triggers specific cell signaling activities (see TABLE) [125,127]. aaRS can also catalyze the synthesis of dinucleoside oligophosphates, and thus indirectly influence many other cell functions such as metal chelation and cell wall metabolism.

Evolutionary divergence from aa-synthetases has provided additional functions for “paralogs”, which resemble the aa-synthetases in sequence and structure, and sometimes also in function. These paralog functions have been reviewed by Pang, et al. [127] and Schimmel, et al., [128]. “For instance, the *E. coli* YadB protein resembles GluRS and attaches glutamate to a queuosine base (position 34) at the first anticodon position of tRNA^Asp^ [129]. This modification expands and alters the tRNA decoding of codons [129,130]. HisZ, a paralog of HisRS, is one component of the enzyme catalyzing the first step of histidine biosynthesis (Sissler, et al., 1999” [131]. Like some of the tRNA synthetases, paralogs also carry out cyclodipeptide synthase activities [132,133].

Paralogs also play critical roles in translation by replacing missing amino acid tRNA synthetases. It is a common misconception is that all living organisms have a complete complement of 20 aaRS corresponding to each of the essential amino acids. In fact, 59% of organisms have less than the required 20, and 22% have more than the required 20, leaving only 19% with the exact complement that is often stated in textbooks. In organisms with both too few and too many aaRS, function is supplied by paralogs that are often able to modify one amino acid into another [134]. Many of these paralogs have taken on additional, non-ribosomal functions that are summarized in Table 7, which include amino acid synthesis, metabolic synthesis, polymerase activity, and stress and immunological functions [128,135,136,137,138]. Notably, the human 50S ribosomal protein L2 and *Mycobacterium* lysyl-tRNA synthetase are very similar [139], suggesting that ribosomal proteins and tRNA synthetases may have common origins—another observation that is consistent with a ribosome-first origin of cellular function, but which could also emerge from a protein-first theory as well.

### 2.6. Non-Canonical Functions of RNA Polymerases

Finally, it is worth noting that the ribosome-first theory of cellular evolution requires that the rRNA be able to replicate, and therefore, that products of rRNA genes must include RNA polymerases. We have demonstrated that key active site sequences of RNA polymerases do appear in various rRNA reading frames in a previous paper [6]. In keeping with the predictions made above that ribosome-related proteins should play extra-ribosomal roles in the formation of the first genomes, it is important to remember that RNA- and DNA-polymerases evolved from common ancestral enzymes, containing a single highly conserved peptide at their catalytic core and which seems to be able to polymerize both types of polynucleotides [140,141,142]. Therefore, a self-replicating ribosome encoding an RNA-polymerase catalytic core could have given rise to the first DNA polymerases, thereby facilitating the transition to a DNA-encoded genome. Even beyond that necessary evolutionary step, however, RNA polymerases have been found to play at least two additional roles in modern cells. One is gene silencing and transcription regulation [143,144], and the other is in myeloproliferation and hematopoiesis [145]. Given the wide range of functions taken on by other ribosome-related molecules, we predict that the polymerases will also be found to have additional functions.

### 2.7. Theory Testing: How Well Do These Data Fit Other Possible Explanations of Genome Evolution?

Obviously, the data summarized above argues strongly for rRNA-containing genetic information that is expressed as translated proteins that are unrelated to ribosome function, and that rRNA-like and tRNA-like genetic sequences occur far more frequently than is either necessary or expected if rRNA and tRNA contain only structural, but no genetic, information. These finding raise the question of how much more frequent is such rRNA-like genetic information than one would expect. Here we attempt to address this question by comparing the frequency with which rRNA-like sequences appear in the *E. coli* K12 genome, as compared with the frequency that one would expect on the basis of a set of randomly generated rRNA-like sequences, and on the basis of a set of protein-encoding mRNAs of equivalent lengths.

**Figure 1:**
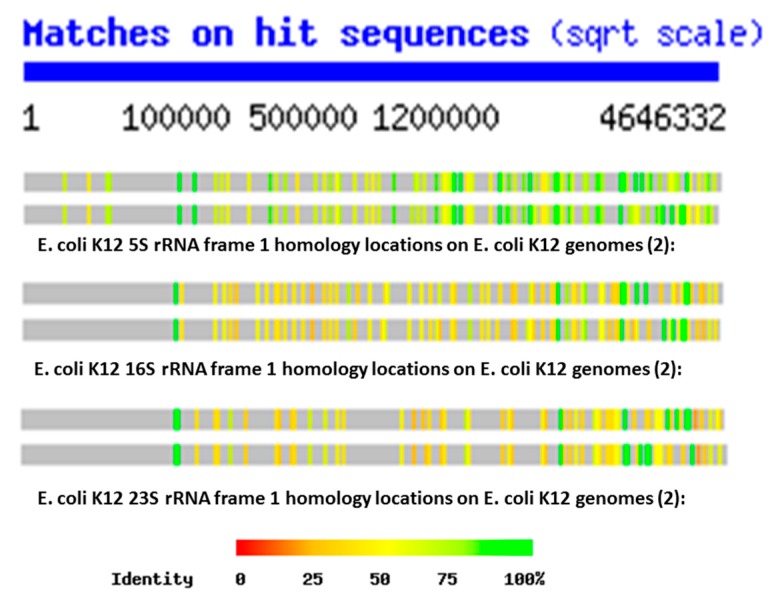
Locations of 5S rRNA, 16S rRNA, and 23S rRNA frame 1 homologies within two strains of *E. coli* K12 genomes. Note that the scale is non-linear. Homology locations are not identical between the 5S, 16S, and 23S rRNAs, demonstrating that the sequences probably have independent origins. Similarly, the homology locations are not identical even between two closely related *E. coli* K12 strains, suggesting that these sequences are subject to reorganization processes within these genomes.

**Figure 2:**
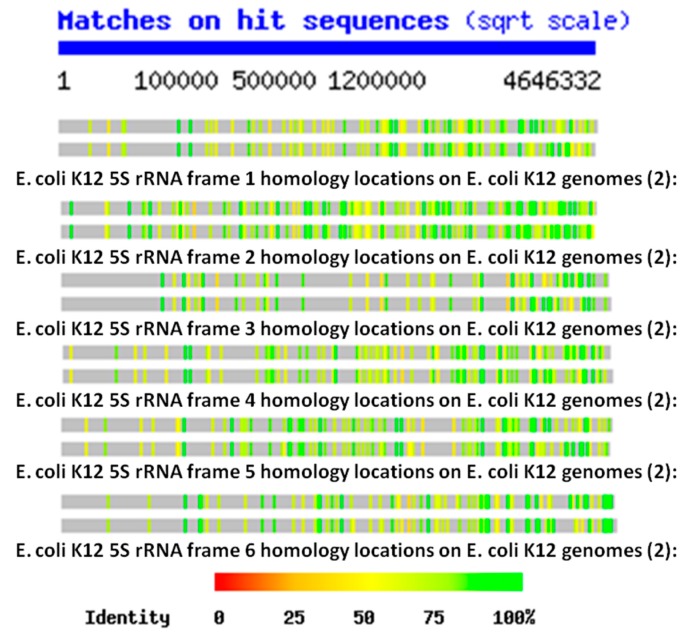
Locations of the six frames of 5S rRNA homologies within two strains of *E. coli* K12. Note that the scale is non-linear. As in the previous Figure, these homology locations differ between the two strains indicating that they are subject to reorganization processes. Notably, the locations also differ considerably from one reading frame to another, demonstrating that the search algorithm is identifying non-overlapping sequences.

The key data for testing these three possibilities are Figure 1 and Figure 2, showing the distributions of rRNA-encoded proteins from all reading frames that are present in the *E. coli* K12 genome. These figures show that, on average, the *E. coli* K12 genome contains hundreds of copies of some version (translated in the six possible reading frames of the RNAs) of each of the 5S, 16S and 23S rRNAs. This observation is confirmed in the Tables below as well, and the data are consistent with the findings of Mauro and Edelman (1999) and many other studies of the ubiquity of rRNA-like sequences in the expressed proteins reviewed above. Four notable features of the locations of the rRNA homologies emerge from these plots. One is that the homology locations differ quite obviously between the 5S, 16S, and 23S homologies, indicating that they probably have independent origins (Figure 1). Secondly, the locations also differ from one reading frame to another (Figure 2), demonstrating that the homology search program is not merely picking up different versions of the same sequence. Third, the locations of the homologies differ even between closely related strains of *E. coli* K12 (Figure 1 and Figure 2), indicating that they are subject to rearrangement processes. Finally, the homology locations, especially when averaged over all six possible reading frames, are more or less randomly distributed across the entire *E. coli* genome so that any region of 10,000 or so base pairs is very likely to have several sequences homologous to at least one of the six reading frames of the 5S, 16S, or 23S rRNAs.

**Figure 3:**
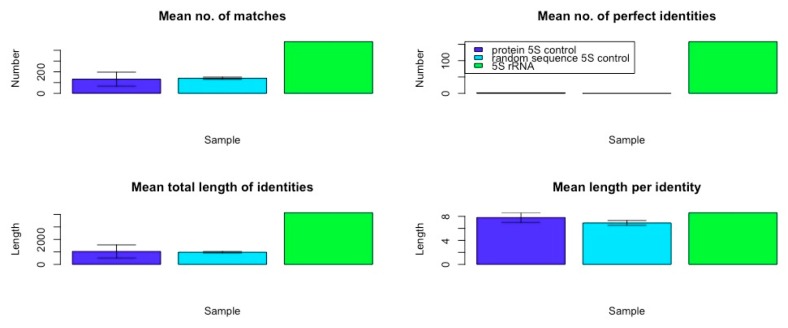
Comparison of 5S rRNA variables with matched protein mRNA and randomized RNA controls (original data in Tables above). Error bars show SD. While the mean length of the matches found in each group was very similar, the number of total matches, the number of identical amino acids found within those matches, and the number of exact matches found to the 5S rRNA was very significantly greater than those found for the proteins’ control mRNA and randomized RNA sequences tested.

**Figure 4:**
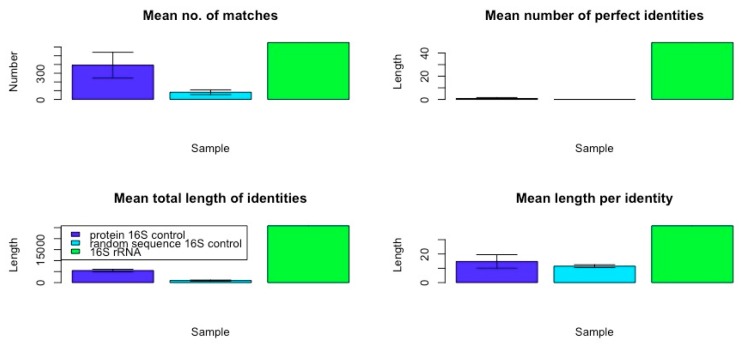
Comparison of 16S rRNA variables with matched protein mRNA and randomized RNA controls (original data in Tables above). Error bars show SD. The mean number of matches found within the *E. coli* genome was greatest for the 16S rRNA, as was the mean number of perfect identities, length of the sequences with identical sequences and the number of identical amino acid matches found within each match was significantly greater for the 23S rRNA than for the mRNA of the proteins controls or the randomized RNA sequences.

**Figure 5:**
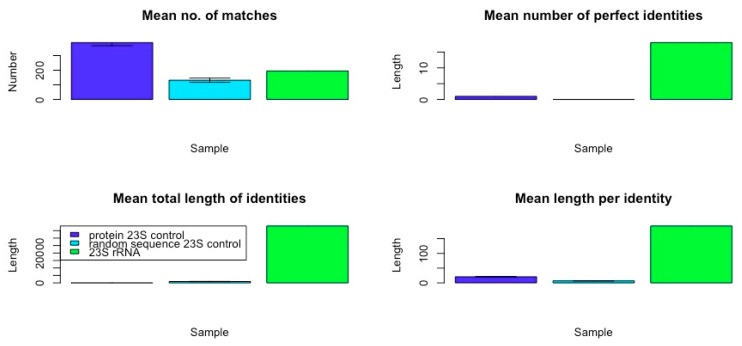
Comparison of 23S rRNA variables with matched protein mRNA and randomized RNA controls (original data in Tables above). Error bars show SD. Although the mean number of matches found within the *E. coli* genome was greatest for the protein controls, the mean number of perfect identities, length of the sequences with identical sequences and the number of identical amino acid matches found within each match was significantly greater for the 23S rRNA than for the mRNA of the protein controls or the randomized RNA sequences.

The data resulting from a comparison of the frequency and qualities of rRNA in the *E. coli* genome confirm that ribosomal RNA-like sequences appear in the genome at far higher frequencies with much longer and more similar matches than do matches to randomized RNA sequences of equal length, or matches from *E. coli* protein mRNA of equal length or randomized RNA sequences. Table 8, Table 9 and Table 10 and Figure 3 summarize the data comparing the frequency and quality of 5S rRNA homologies within the *E. coli* K12 genome with data from a randomly selected set of protein mRNA of equivalent length and a set of randomly generated RNA sequences. Table 11, Table 12 and Table 13 and Figure 4 summarize the data comparing the frequency and quality of 16S rRNA homologies within the *E. coli* K12 genome with data from a randomly selected set of protein mRNA of equivalent length, and a set of randomly generated RNA sequences. Table 14, Table 15 and Table 16 and Figure 5 summarize the data comparing the frequency and quality of 23S rRNA homologies within the *E. coli* K12 genome with data from a randomly selected set of protein mRNA of equivalent length and a set of randomly generated RNA sequences. 

In general (with the exception of the 23S protein matches) the number of rRNA homologies was significantly greater than the number of mRNA and random RNA matches. The number of matches differed by sample type (protein control, random control, or rRNA) for the 5S rRNA (ANOVA, F2,7 = 18.7, *p* = 0.002), for the 16S (ANOVA, F_2,9_ = 17.76, *p* = 0.001), and for the 23S (ANOVA, F_2,6_ = 187, *p* = 2 × 10^−5^). However, for the 23S samples, it was the protein control that had the greatest number of matches (see Figure 5). The random RNA sequences had no identical matches with the *E. coli* genome, where “identity” is defined as at least 90% identical amino acids over a sequence comprising at least 90% of the length of the RNA used as the search string. Thus, a 120 base pair (5S rRNA) would have to match a continuous sequence of 108 base pairs, with at least 90% identical amino acids in the *E. coli* genome, to satisfy the “identity” classification. Each protein-derived mRNA sequence had one (and occasionally two) identities with the genome, as would be expected from the fact that these proteins are encoded in the genome. The rRNA sequences, in contrast, had dozens of identical matches scattered throughout the genome, as shown in Figure 1 and Figure 2. These data argue against a random or protein origin of the genome while supporting an rRNA origin.

Additional evidence for an rRNA origin was found in the quality measures derived from the homology searches. With the exception of the 5S rRNA matches, which were no longer on average than those derived from mRNAs or random RNA sequences, rRNA matches were significantly longer than the controls (Figure 3, Figure 4 and Figure 5). Mean identity length did not differ across sample type (protein control, random control, or rRNA) for the 5S rRNA (ANOVA, F2,7 = 2.938, *p* = 0.129). Mean identity length differed significantly by sample type for 16S rRNA (ANOVA, F_2,9_ = 26.09, *p* = 0.0003), and for 23S (ANOVA, F_2,6_ = 97,253, *p* = 3.35 × 10^−12^).

Moreover, the number of identical amino acids found within these longer matches was also significantly greater, so that these longer matches were also better matches (Figure 3, Figure 4 and Figure 5). The number of perfect identities differed by sample type (protein control, random control, or rRNA) for the 5S rRNA (ANOVA, F2,7 = 20,545, *p* = 3.11 × 10^−12^), 16S (ANOVA, F_2,9_ = 3070, *p* = 5.4 × 10^−11^), and 23S (ANOVA, F_2,6_ = 1.5 × 10^33^, *p* < 2 × 10^−16^). The data derived by looking at amino acid similarities rather than identities was virtually identical from a statistical perspective; adding the similar amino acids within each sequence to the identities merely increased the quality of the matches without further differentiating the rRNAs from the controls, and so these data have not been displayed.

The average length of the rRNA matches is seven times that of the random RNA matches, and the number of identical amino acids within those matches is 14 times as many as in the random RNA matches. The mean length of identities in matches differed by sample type (protein control, random control, or rRNA) for the 5S rRNA (ANOVA, F_2,7_ = 23.54, *p* = 0.001), for 16S, (ANOVA, F_2,9_ = 1470, *p* = 5.43 × 10^−11^), and for 23S (ANOVA, F_2,6_ = 24,496, *p* = 1.05 × 10^−10^). Altogether, the rRNA matches account for 11 (16S rRNA) to 19 (23S rRNA) times the amount of the *E. coli* genome that is recognized as similar, as do the random RNA matches or the protein-derived mRNA matches. rRNA sequences are clearly highly over-represented compared with either control group.

In sum, rRNA sequences are significantly more likely to match the genome than are sets of mRNA derived from *E. coli* proteins of similar lengths. The average length of an rRNA match is six times that of an mRNA match (for 16S- and 23S-length sequences) and, as with the random sequences, the rRNA matches are significantly more likely than the mRNA matches to encode more amino acid identities so that overall the rRNAs encode four to six times as many identical amino acids within their matches (and this, in spite of the fact that BLAST yielded three times as many mRNA matches than 23S rRNA matches). Most significantly, while a typical mRNA sequence had 95% or more identity over its entire length (an “identity”) with only one genome sequence (as would be expected for matching its gene), the rRNA sequences yielded dozens of exact homologies. Thus, while protein sequences generally occur in only one copy in one reading frame, rRNA sequences occur in many copies in multiple reading frames. No random sequence in our study yielded any exact homologies. Protein mRNA matches are more similar to random sequence matches than to rRNA matches.

Overall, this statics-based similarity investigation into the frequency and qualities of rRNA-like, mRNA-like and randomized RNA-like sequences in the *E. coli* genome confirms that rRNA-like sequences occur very significantly more often than one would expect by chance, and far more often than mRNA duplications do. Indeed, it is possible to calculate the total number of base pairs involved in the similarities revealed in the Tables above, which yields the surprising result that, although the 5S, 16S, and 23S rRNAs, which total 4566 bp and account for only 0.098% of the 4,646,332 bp of the *E. coli* K12 genome, similar sequences (at E = 10 in the BLAST parameters) are present in sufficient copies (in all six reading frames) to account for 1/20 or 5% of the genome (240,171 bp) with quite high fidelity. This compares with 1/250 or 0.4% of the genome matching randomized RNA sequences (18,702 bp) and about 1/70 or 1.3% matching random mRNA (protein-encoding) sequences (66,768 bp), but with far less fidelity than for the rRNA sequences in terms of percent identities within each match. Thus, our hypothesis that rRNA may have been the basis for the first cellular genomes is supported while alternative theories such as a protein-world origin for the genome, or genome-before-ribosome, are not.

## 3. Discussion

**Figure 6:**
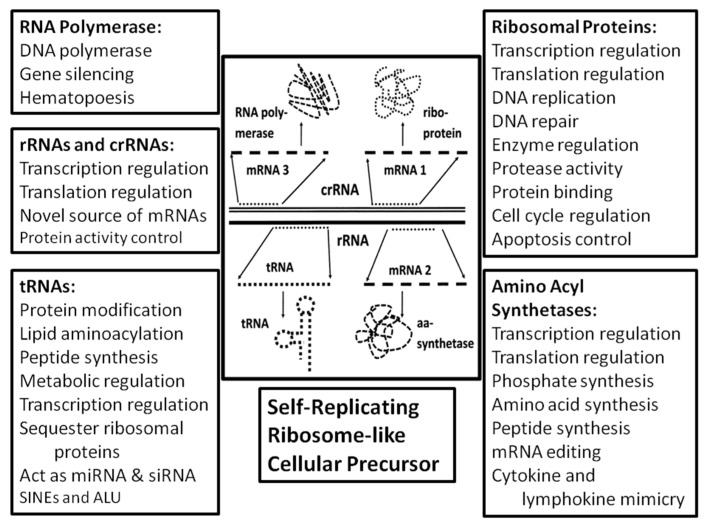
A summary of the evidence for pleiofunctionality in rRNA-related molecules supporting the hypothesis of a self-replicating ribosome. rRNA = ribosomal ribonucleic acid (only one rRNA is shown though there are three in the ribosome, the 5S, 16S, and 23S). crRNA = complementary strand of rRNA posited to exist to make possible rRNA replication in self-replicating ribosome. The crRNA would also act as a messenger RNA (mRNA), or act itself as a source of transcription to produce mRNAs (see Root-Bernstein and Root-Bernstein, 2016; 2017). tRNA = transfer ribonucleic acid. aa-synthetase = amino acyl synthetase, which loads amino acids onto tRNA. Ribo-protein = ribosomal proteins (see Table 3, Table 4, Table 5 and Table 6). Boxes summarize the data from Table 1, Table 2, Table 3, Table 4, Table 5, Table 6 and Table 7 and the related text.

To summarize, we have reviewed existing studies that demonstrate evidence that ribosome-related RNAs are present in unexpectedly large quantities in all genomes, and that these rRNAs, tRNAs and ribosome-related proteins play a wide range of extra-ribosomal, plieofunctional roles in modern cellular systems. Large numbers of rRNA-like sequences in both prokaryotes and eukaryotes are transcribed as polyadenylated mRNAs and, in many cases, translated into functional proteins. rRNA- and tRNA-like transposons are known to make up a significant proportion of all genomes, which may provide a mechanism by which ribosome-related genes have proliferated within cellular genomes. rRNAs and tRNAs and their fragments play multiple extra-ribosomal roles as regulators of transcription and translation, as metal chelators, by carrying out various forms of non-ribosomal protein synthesis, as mRNAs, and in cell signaling functions. Nearly all ribosomal proteins are known to perform at least one, and often multiple, extra-ribosomal functions ranging from autologous regulation of their own mRNAs by means of binding to them, to control of replication, transcription and translation, participation in polyphosphate synthesis and metabolism, and carrying out various enzyme functions. Aminoacyl tRNA synthetases similarly play a wide range of extra-ribosomal functions that include replication, transcription, and translation regulation, polyphosphate metabolism, dipeptide synthesis, and cell signaling. These synthetases have given rise to a large number of paralogs that carry out additional enzymatic functions including amino acid synthesis, RNA editing, and energy metabolism (Figure 6). Thus, ribosome-related molecules are capable of carrying out most key cellular functions as predicted on the basis of the ribosome-first theory of cellular evolution. In addition, we have demonstrated here for the first time that although rRNAencoding genes make only a few hundredths of a percent of the *E. coli* genome, rRNA-like sequences constitute no less than 1/20th or 5% of the *E. coli* genome. This prevalence is many times greater than would be predicted if the genome evolved from random sequences of RNA or from divergence from protein-encoding genes. Note further that our analysis did not investigate the occurrence of tRNA-like sequences, SINES or LINES, which the literature cited above suggests may account as much as 10–20% of many genomes. In short, our analysis suggests why ribosome-related genes are the core of the approximately 100 orthologous genes conserved across all species that make up the Universal Gene Set of Life (UGSL) [1,2,3].

Consider these data in light of the three possible scenarios outlined in our Introduction. A genome-first approach does not fit the evidence for multiple ribosomal molecule function at all. In a genome-first approach, translation is added to cellular functions after an RNA-world has already evolved autonomously, without feedback from protein–world interactions. rRNA and tRNA genes would have been selected without regard for protein function and so have no predictive value for other cellular functions. Additionally, if ribosomes evolved separately from an RNA- or DNA-based genetic world, then one would expect to find, as current dogma maintains, that ribosomal genes have no functions other than to encode rRNA and that rRNA contains no genetic information. These predictions are not borne out by the evidence gathered here.

A metabolism-first approach to cellular evolution runs into a different conundrum: the possibility that rRNAs, tRNAs, and ribosomal proteins all evolved separately for non-translational functions, and then reached a critical point, at which they suddenly integrated to perform translation functions seems not only miraculous in its implausibility, but leads to the inherent contradiction that cellular life evolved without the means to reliably produce the proteins required to make it possible. Thus, while there is every reason to predict that ribosomal proteins would have non-ribosomal functions in a metabolism-first scenario, the difficulties in evolving a ribosome in the first place produce significant barriers to a metabolism-before-genes model, and our evidence suggests that a protein-first model of genome evolution is only marginally better than a totally random origin of genome sequences.

**Figure 7:**
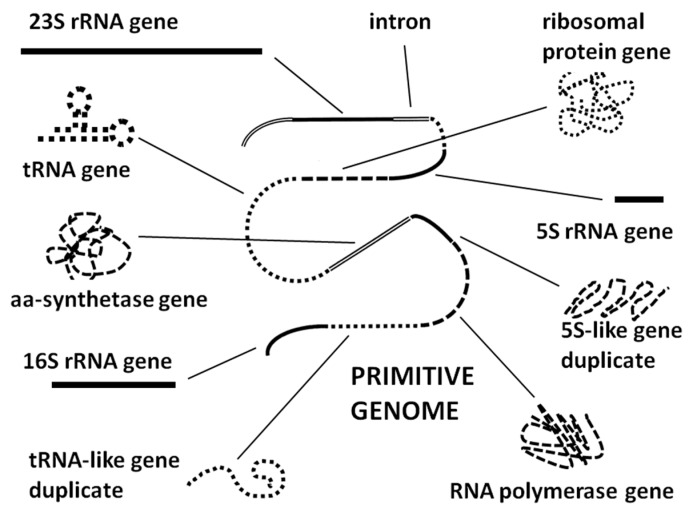
Schematic model of how RNAs perform various ribosomal functions and/or encoding various ribosome-related proteins may have become ligated to form the first genome. The visual formalisms are the same as in Figure 6 and illustrate the idea that “genes” that may have existed as overlapping sequences in a variety of reading frames within a self-replicating set or rRNAs may have been separated out into a single reading frame for better functional control. This schema does not identify whether the original primitive genome was constituted of RNA or DNA (though RNA seems likely if a ribosomal origin is correct) but given a common origin for RNA and DNA polymerases (see text) the existence of either one could have provided the basis for the other. It is also important to remember that SINEs and other insertion-facilitating RNAs most likely evolved from tRNAs or that they have common ancestors (see text), so that molecular mechanisms of genome growth would have been present from the outset in a ribosome-first scenario.

The ribosome-first theory is the only current theory that is consistent with all data gathered here and the only one that explains the co-evolution of genetic information and translation function from the outset. The ribosome first theory is consistent with the prebiotic evolution of an RNA–protein world [12,146,147,148,149,150], thereby integrating observations that both RNA species (“RNA world” scenarios [11,150,151,152,153,154,155,156]) and amino acids (“protein world” scenarios [157,158]) were present simultaneously from the outset. More importantly, the ribosome-first theory is consistent with multiple taxonomic studies that all place the origin of functional ribosomes earlier than the last universal common ancestor (LUCA) and therefore, with high probability, prior to the inception of cellular life itself [159,160,161,162,163]. If the cell evolved, at least in part, to optimize preexisting ribosomal functions by providing a protected, homeostatic environment with a ready and steady supply of precursors and energy, then the incorporation of rRNA sequences into the very fabric of the cellular genome in multiple copies and multiple reading frames becomes logically comprehensible. Ribosomal RNA would already function like a small genome encoding the ribosome and its attendant tRNAs and proteins. Having already optimized the information content of their “genomes” in order to exist as “free-living” selfish agents, ribosome-like entities would have been among the primary donors of genetic material to emergent cellular life. Such cellular life would have vastly increased the metabolic efficiency, stability, and probability of successful replication of such ribosome-like entities by accumulating necessary resources for the ribosome and providing an optimized environment for its function. As part of this process, the cellular chromosome would have “unpacked” the highly integrated information within the rRNAs (in which every reading frame encodes functional protein segments as well as serving as mRNAs and tRNAs) and transferred these separate functions to the cellular genome. As separate genes, this information would be more readily accessible for transcription and translation than when “packed” as overlapping sequences; it would be more amenable to control through the incorporation of promoter and repressor sequences; and it could be stored in the much more stable form of DNA genes. The evolution of DNA-encoded genes may have provided a high degree of stability to a previously highly varying ecology of self-replicating ribosome-like entities, and selection for stabilization of the translation function of these ribosome-like entities at the expense of their evolvability. Evolutionary processes would have replicated and produced divergences in the DNA-encoded genetic sequences and recombined these sequences with genetic material from other sources to yield molecular novelties so that the various reading frames of the rRNAs would have found novel uses throughout the new cellular genome (Figure 7). By building up the cellular genome from already tried-and-true interactive modules, the integrity of the cellular interactome would have been assured as cell functions expanded and diversified. The present-day ribosome would then have been able to divest some of its functions by off-loading them to the cellular genome in order to optimize translation efficiency.

This is not to say that the ribosome-first theory of cellular origins explains all aspects of cellular function or genome structure. It is evident from the list of functions that are associated with ribosomal molecules that these do not include critical aspects such as glucose metabolism (e.g., the origins of the Krebs and Calvin cycles), nor does it explain the origins of other key cellular organelles such as acidocalcisomes and cell membranes that are found in all cells. The origins of these structures should also attract serious investigation and mechanisms by which cells evolved to integrate them with ribosomes and genomes need to be explored.

We suggest in conclusion that our modular approach to evaluating the evolution of such bacterial genomes reveals a fundamental mechanism by which complexity and diversity resulted from mixing and matching simple, already-proven, functionally interactive, genetically encoded protein fragments. By using such pre-selected modules as building blocks for more complex genes and proteins, the number of different permutations required to find new functional elements was minimized, the probability of retaining functionality greatly increased, and the interactivity of these elements maximized. The specific results of our analysis can therefore been seen as a concrete example of an evolutionary self-assembly principle proposed by Simon [164], who demonstrated theoretically that complex systems are much more likely to evolve from interactive, semi-stable modules than from the random assortment of components that evolve independently of each other (see also [165,166]). Our approach also suggests new avenues for research on the origins of cellular life. Looking backwards to the evolution of self-replicating ribosome-like entities, the transposon-like functions of so many tRNA- and rRNA-like genes suggests that ribosomes themselves may have evolved from a distributed ecology of virus-like particles capable of rapid evolution through gene swapping and rearrangement. The ribosome would have been a critical step in integrating this distributed, co-evolving set of molecules into a stable, efficient replication-transcription–translation system.

## 4. Materials and Methods

Escherichia coli K12 rRNA (5S, 16S, and 23S) sequences were obtained from http://ecoliwiki.net/colipedia/index.php/16S_rRNA:Gene_Product%28s%29. Each RNA was translated into its six protein translation reading frames using a translate tool (https://web.expasy.org/translate/). The protein sequences resulting from the six reading frames of the 5S, 16S, and 23S rRNA were compared with the *E. coli* K12 genome in the Nucleotide database using TBLASTN (version 2.2.31+), which searches protein sequences against the nucleotide sequence of the genome thereby permitting all possible reading frames to be examined. Parameters were set as follows: E = 10, the low complexity filter on, no gapped alignments, and 100 of the best scoring sequences and alignments to show. The resulting homologies were recorded for each frame in each rRNA sequence, in terms of the overall length of the protein match, how many identical amino acids were present in the homology, and how many similar amino acids were present. The same procedure was performed with a series of mRNA from control proteins of the *E. coli* K12 genome accessed from the U. C. Santa Cruz microbial genome list: http://microbes.ucsc.edu/lists/eschColi_K12/refSeq-list.html. The rRNA sequences and the proteins resulting from their six reading frames are provided in Supplementary Table V. Three sets of mRNA encoding *E. coli* proteins were explored for comparison, one set of five averaging about 120 bp in length to mimic the 5S rRNA; as second set of five averaging about 1500 bp in length to mimic the 16S rRNA, and a third set of two averaging about 2900 bp in length to mimic the 23S rRNA. The mRNA sequences encoding the proteins are provided in Supplementary Table W. The results for each set of five proteins were averaged for comparison with the *E. coli* K12 rRNA results obtained above. The proteins and their identifiers are listed in Table 1. The same procedure was performed one more time with three sets of further control RNA sequences generated using an RNA random sequence generator (http://www.molbiotools.com/randomsequencegenerator.html). Lengths were matched to the lengths of the 5S rRNA (120 bp), 16S rRNA (1500 bp) and 23S rRNA (2900 bp). The random sequences are provided in Supplementary Table X.

Ribosomal RNA-encoded protein location and copy number in *E. coli* K12 genome was determined using a function that has, unfortunately, since been removed from the program during a recent upgrade. Each reading frame for each rRNA (5S, 16S, and 23S rRNA) was compared using the nblast function at the Swiss Institute of Bioinformatics website (http://www.expasy.org) function with two *E. coli* K12 genomes (“ECOLI”, 2 versions: U00096 Escherichia coli MG1655 Chromosome and AP009048 *Escherichia coli* ATCC 27325 Chromosome). The resulting data did not identify specific protein homologies but provided information about where the sequences appeared within the genome and how many copies of that particular rRNA-encoded protein were present in the genome. (The same kind of data can still be obtained, but must now be graphed onto the genome “by hand” using the bp location data provided by the search).

Statistics: ANOVA using the “aov” command in R version 3.3.3 (https://cran.r-project.org/bin/macosx/).

## Figures and Tables

**Table 1:** Non-canonical functions of transfer RNAs (tRNAs), their fragments, and mimics. Table based mainly on references [53,54,55,56,57,58,59,60,61,62]. See text for additional references. miRNA = microRNA; siRNA = small interfering RNA.

Non-Canonical tRNA Properties	Molecules Involved	Functions
**Modification of Macromolecules**		
Lipid aminoacylation	Phosphatidylglycerol synthetases	Protects against some antibiotics
Enzymatic modification of peptidoglycans	MurM, Fem aminoacyl transferases	Modifies peptidoglycan synthesis in cell wall; alters protein binding
Enzymatic modification of amino terminus of proteins	Leucyl/Phenylalanyl transferases; Arg-tRNA transferase	Targets proteins for degradation
Enzymatic modification of peptides	AlbC, PacB, VlmA, DhpH-C peptidyltransferase	Intracellular signaling; Metal chelation; Antibiotic formation
**Synthesis of Small Metabolites**		
Dipeptide synthesis	Diketopiperazines	Metal chelation
Tetrapyrrole synthesis	Heme; Chlorophyll	Energy transduction
Aminoacylation of tRNA	Gln; Asn; fMet; Cys; Sec	Makes up for missing tRNA
**Regulatory Processes**		
Bind directly to ribosome to modify activity	Deacylated tRNA	Stringent response: Form alarmones, ppGpp and pppGpp
DNA replication initiation	ColE1-type plasmids	Control of plasmid replication
Riboswitch control	Operons ; T-box	Transcription attenuation
Metabolism	Siderophores; Virulence factors	Stress responses; Aerobic/respiratory metabolism; Gene expression
Stress responses	Proteins and polysomes involved in siRNA binding & miRNA binding	Translation regulation
**tRNA Mimicry**		
Translation regulation	tmRNA (small transfer-messenger RNA; SsrA or 10Sa RNA) & tmRNA-specific binding protein (SmpB or small protein B).	Rescues stalled ribosomes
“t-elements” (tRNA-like elements) in mRNAs	3′ and 5′ ends of mitochondrial mRNA	Sites for RNAase P and RNAase Z activation of mRNA?
Short Interspersed nuclear elements (SINEs)	Genome (non-coding DNA)	Retrotransposons
Viral tRNA mimics	Viral mRNA	Hijacking translation machinery
**tRNA Fragments**		
tRNA halves	Outer membrane vesicles	Stress responses? Bacterial signaling?
tRNA-derived fragments (tRFs)	Ribosomal proteins; mRNAs; RNA-binding proteins	Interferes w/with peptidyl transferase; Act as miRNAs or siRNAs in post-transcriptional gene silencing; Buffer regulatory RNAs; Sequester RNA-binding proteins

**Table 2:** Non-canonical functions of ribosomal RNA (rRNA) [82,83,84,85,86,87,88].

Non-Canonical rRNA Properties	Molecules Involved	Functions
Transcription regulation	5S rRNA acting on transcription factor for polymerase factor IIIA	Inhibits activation of RNA polymerase III-dependent transcription and related genes
Translation regulation	Ribosomal protein fragments	siRNA and RNAi inhibiting mRNA translation
Permits read-through of termination codons	23S rRNA fragment	Inhibits release factor 2 (RF2)-dependent translation termination
Protein activity control	5S rRNA-RPL5-RPL11 complex	Inhibits the Hdm2 protein which in turn regulates p53
Sources of novel transcripts	5.8S and 28S rRNA fragments	Translated into proteins

**Table 3:** Extra-ribosomal functions of ribosomal proteins of the small subunit of prokaryotic ribosomes (PROK RP). Auto Reg = autologous regulation of ribosomal protein by binding to its own mRNA.

Prok RP	Auto REG	Other Extra-Ribosomal Functions of RP	References
S1	YES	Stimulates RNA polymerase; Phage Qβ replicase	[7,89,90,91]
S2	YES	Regulates rpsB and tsf genes	[7,89]
S3	YES	DNA repair; NF-κB signaling pathway; Apoptosis	[7,92]
S4	YES	Regulates rpsD gene and α operons; Transcription anti-termination; NUS transcription elongation complex	[7,89,90]
S5	YES		[7]
S6	YES		[7]
S7	YES		[7]
S8	YES	Repressor of spc operon	[7,89]
S9	YES	SOS DNA repair system	[7,89,91]
S10	YES	Transcription anti-termination; NUS transcription elongation complex	[7,89,90,91]
S11	YES		[7]
S12	YES	T4 phage RNA chaperone and intron splicing	[7,89,91]
S13	YES		[7]
S15	YES		[89]
S18	YES		[7]
S19	YES		[7]
S20	YES	Inhibition of ornithine and arginine decarboxylase enzymes	[7,89]
S21	YES		[7]
S26	YES		[7]
S28	YES		[7]

**Table 4:** Extra-ribosomal functions of ribosomal proteins of the large subunit of prokaryotic ribosomes (PROK RP). Auto Reg = autologous regulation of ribosomal protein by binding to its own mRNA.

Prok RP	Auto REG	Other Extra-Ribosomal Functions of RP	References
L1	YES		[7,89]
L2	YES		[7]
L3		Transcription anti-termination	[7,89]
L4	YES	Transcription anti-termination; inhibits S10 transcription; regulates RNAase E	[7,89,90]
L6	YES		[7]
L7/12	YES		[7]
L10	YES		[7,89]
L11	YES		[7]
L13		Transcription anti-termination	[7,89]
L14		Phage replication helicase	[7,91]
L19	YES		[7]
L20	YES		[7]
L24	YES		[7]
L25	YES		[7]
L30	YES		[7]
L31	YES		[7]
L32	YES		[7]
L35	YES		[7]

**Table 5:** Extra-ribosomal functions of ribosomal proteins of the small subunit of eukaryotic ribosomes (EUK RP). Auto Reg = autologous regulation of ribosomal protein by binding to its own mRNA.

Euk RP	Auto REG	Extra-Ribosomal Functions of RP	Reference
SA		67 kD Laminin receptor	[93]
S0+S21		Promotes 18S rRNA maturation	[89]
S2		Methylation of Rps2p; Laminin receptor	[95,96]
S3		Identical to apurinic-apyrimidinic DNA endonuclease III; functions as DNA β-lyase; binds NFkB; Activates p53 tumor suppression gene; Cdk2- mediated cell cycle regulation; Inhibits RECQL4 ATPase/helicase; Substrate of Akt mediating neuronal apoptosis and DNA repair; Binds HSP 90/70; Binds the transcription factor CHOP	[89,90,91,95,97,98,99,100,101]
S4		Cysteine protease	[102]
S6	YES	Translation regulator; Replication regulator; Enhances virus replication	[7,95,97]
S7		Binds MDM2; Activates p53 tumor suppression gene; Regulates MAPK; Binds to BCCIPβ	[97,101,103]
S8		Identical to p27, an nuclear oocyte peripheral membrane protein	[91]
S9	YES	Translation regulator	[7,95]
S12	YES	RNA splicing and modification; Karyopherin Kap121-dependent sequestration of ribonucleotide reductase	[7,89,90,104]
S13	YES	Regulates own intron and mRNA splicing	[7,89]
S14	YES	Inhibits own mRNA splicing; Regulates own transcription; Represses CRY2 expression; Activates p53 & Tap73 tumor suppression genes; Inactivates c-Myc	[7,89,90,95,97]
S15		Activates p53 tumor suppression gene	[97]
S19		Apoptosis; Inhibits MIF, ERK, NF-κB; Erythropoiesis via C5a receptor binding	[93,94,97]
S20		Regulates Pol III transcription; Apoptosis; Activates p53 tumor suppression gene	[89,93,95,97]
S26	YES	Developmental processes	[7,89,95]
S27		Activates p53 tumor suppression gene	[97]
S28	YES		[7,90,95]
S30	YES		[7,89]

**Table 6:** Extra-ribosomal functions of ribosomal proteins of the large subunit of eukaryotic ribosomes (EUK RP). Auto Reg = autologous regulation of ribosomal protein by binding to its own mRNA.

Euk RP	Auto REG	Extra-Ribosomal Functions of RP	Reference
L1	YES	Regulates gene expression	[7,95]
L2	YES		[7,90,95]
L3		Cell cycle regulation; Apoptosis	[105]
L4	YES	Regulates nucleolar RNA helicase 2 (Guα); Inhibits virus production	[7,89,90,97]
L5		Binds to MDM2; Activates p53 & Tap73 tumor suppression genes; Inactivates c-Myc; Stimulates aminoacyl tRNA synthases	[91,93,97]
L6		Regulates Pol III gene transcription; Activates p53 tumor suppression gene	[95,97]
L7	YES	Cell arrest; Apoptosis; Co-regulates VDR-RXR nuclear receptor complex	[7,93,101]
L10	YES	Translation regulator; Replication regulator; Binds c-Jun gene; Antiviral activity	[7,90,95]
L11		Regulates PPARα gene transcription; Activates p53 & Tap73 tumor suppression genes; Inactivates c-Myc	[89,97]
L12	YES		[7,90]
L13a		Regulates inflammatory gene expression and ceruloplasmin mRNA translation (GAIT complex); Anti-viral immunity; Binds to glyceraldehyde-3-phosphate dehydrogenase	[89,97,106,107]
L21		Apoptosis	[93]
L22		Binds histone H1; Binds EBV small RNA; modulates splicing of the pre-mRNA encoding smad2 (morphogenesis); Hematopoesis; Binds to casein kinase 2α	[90,93,108,109,110]
L23		Activates p53 tumor suppression gene; Binds MDM2	[97]
L26		Activates p53 tumor suppression gene; Binds MDM2	[97]
L27		Apoptosis; Developmental control	[93,111]
L30	YES		[7,90]
L31		Apoptosis	[93]
L32	YES		[7,95]
L33A		Inhibits GCN4 translation	[95]
L37		Activates p53 tumor suppression gene	[97]
Asc1		Cell-wall integrity; Iron homeostasis; Energy metabolism	[95]
P-prots		Regulates phosphate transporters and phosphatases	[95]
P0/LP0		Endonuclease; DNA repair	[89,91,93]
P2		Iron-binding protein	[91]
RACK1		Receptor of activated C kinase (signal transduction)	[90]

**Table 7:** Non-canonical functions of tRNA synthetases and their paralogs. Table based on [117,118,119,120,121,122,123,124,125,126,127,128,129,130,131,132,133,134,135,136,137,138,139].

Non-Canonical Properties of Aminoacyl tRNA Synthetases (aa-RS)	Molecules Involved	Functions
**Metabolism**		
Lysyl-tRNA synthetase	Nucleosides	Polyphosphate synthesis
**Regulation**		
Alanyl-tRNA synthetase	Ala-tRNA synthetase gene operon	Autogenous regulation of transcription
Asparaginyl-tRNA synthetase	FGF-2 induced osteoblast growth	Regulates anti-apoptotic APK/Atk signalling
Leucyl-tRNA synthetase	Group I introns, mRNA	Splices group 1 introns
Threonyl-tRNA synthetase	Thr-tRNA synthetase mRNA	Autogenous regulation of translation
Tyrosyl-tRNA synthetase	Group I introns, mRNA	Splices group 1 introns
**Signaling**		
Asparaginyl-tRNA synthetase	CXCR1, CXCR2, ERK1, etc.	Activates CCR5 & IL-8 receptors CXCR1 and CXCR2; MAP kinases
Glycyl-tRNA synthetase	Cadherin (CDH6)	Binding to CDH6 (cadherin) releases suppressed phosphatase 2A (PP2A) that dephosphorylates activated ERK
Histidyl-tRNA synthetase	CCR5	Chemokine: CCR5 activation
Tryptophyl-tRNA synthetase	DNA-PKcs, PARP-1	Bridges DNA-PKcs to PARP-1 to link IFN-γ and p53 signaling
**Release of following from MM-aaRS complex:**	**Macromolecular aa-RS Complex (MM-aaRS):**	**Results in the following activity:**
LysRS-II	Complex of ArgRS, GlnRS, IleRS, LeuRS, LysRS, MetRS, AspRS, Glu-ProRS, AIMP1, AIMP2, AIMP3	Inflammatory cytokine (Extracellular) Viral assembly (Plasma membrane) Transcriptional control (Nuclear)
Glu-ProRS	Ditto above	Translational silencing, e.g., VEGF
GlnRS	Ditto above	Anti-apoptosis
MetRS	Ditto above	rRNA transcription
IleRS	Ditto above	Autoimmune response
AIMP1 (aminoacyl-tRNA synthetase interacting multifunctional protein 1) (p43)	Ditto above	Cytokine activation of macrophages
AIMP2 (p38)	Ditto above	Degradation of FBP (transcriptional activator of *c-myc*)
AIMP3 (p18)	Ditto above	p53 induction and DNA repair by activating nuclear ATM/ATR
**Fragments**		
Lysyl-tRNA synthetase	Proteolytic internal fragment of Lys-RS	Cytokine-like domain that functions in chemotaxis
Tryptophyl-RS	Trp-RS C-terminal	Up-regulates TNF-alpha
Tryptophyl-RS	Trp-RS N-terminal	Mimics interleukin-8 functions
Tyrosyl-RS	Tyr-RS C-terminal	Up-regulates TNF-alpha
Tyrosyl-RS	Tyr-RS N-terminal	Mimics interleukin-8 functions
**Paralogs**		
AlaX, paralog of Ala-RS	tRNA	Trans-editing of mischarged tRNA
AlbC, paralog of TyrRS & TrpRS	Phenylalanine, Serine	Cyclodipeptide synthase
AsnA, paralog of Asn-RS	Asparagine	Asparagine biosynthesis
Arc1p, paralog of Met-RS	mRNA	RNA editing
BirA, paralog of Ser-RS	Biotin	Biotin repressor
DNA pol γ, paralog of Gly-RS	Polynucleotides	DNA polymerase
EMAP II, paralog of Tyr-RS	Cytokine receptors	Cytokine
GCN2, paralog of His-RS	Proteins, Histidine	Protein kinase, Histidine biosynthesis
HIsZ, a paralog of His-RS	Histidine	Histidine biosynthesis
LysK, paralog of LysRS	Lysine	Lysine biosynthesis
PheX, paralog of Phe-RS	?	Undetermined
Polyβ, paralog of GlyRS	Polynucleotides	DNA polymerase
PoxA, paralog of LysRS	Elongation factor P	Modifies EF-P
PoxA/GenX, paralog of Lys-RS	Pyruvate	Pyruvate oxidase
ProX, paralog of Pro-RS	tRNA	Trans-editing of mischarged tRNA
Slimp, paralog of mito SerRS	RNA binding	Regulates rRNA, COX2 & 3 mRNA
Trbp111, paralog of Met-RS	mRNA	RNA editing
YadB, or GluX, a paralog of Glu-RS	Adds glutamate to first anticodon position of tRNA-Asp	Stress response
Ybak, paralog of ProRS	Cys-tRNA(Pro)/Cys-tRNA(Cys) deacylase	Trans-editing of mischarged tRNA
YvmC-Blic, class Ic aaRS paralog	Cyclodileucine	Non-ribosomal peptide synthesis

**Table 8:** 5S rRNA protein control sequences utilized to test the frequency and quality of homologous sequences in the *E. coli* K12 genome. Sample numbers identify the mRNA of control proteins of the *E. coli* K12 genome accessed from the U. C. Santa Cruz microbial genome list: http://microbes.ucsc.edu/lists/eschColi_K12/refSeq-list.html. See text for definitions of “identities”, “similarities”, etc.

Protein 5S Control	Count Data Total, Sum of Six Reading Frames		Sums across All Matches			Means across All Matches		
Sample	N^o^. Matches	N^o^. Perfect Identities	Total Length of Matches	Total Number of Similarities	Total Number of Identities	Mean Match Length	Mean Similarity Length	Mean Identity Length
b4626	83	2	632	568	550	7.6	7	6.6
b0466	241	1	3325	2326	1886	13.8	9.6	7.8
b4631	83	1	948	791	672	11.4	9.5	8.1
b4509	127	3	1545	1234	1101	12.1	9.7	8.7
b4515	127	1	1428	1150	990	11.2	9.1	7.8
mean	132.2	1.6	1575.6	1213.8	1039.8	11.2	8.9	7.8
SD	64.7	0.9	1044.9	677.7	523.8	2.3	1.1	0.8

**Table 9:** 5S rRNA random control sequences utilized to test the frequency and quality of similar sequences in the *E. coli* K12 genome. See text for definitions of “identities”, “similarities”, etc.

Random Sequence 5S Control	Count Data Total, Sum 6 Reading Frames		Sums across All Matches			Means across All Matches		
Sample	N^o^. Matches	N^o^. Perfect Identities	Total Length of Matches	Total Number of Similarities	Total Number of Identities	Mean Match Length	Mean Similarity Length	Mean Identity Length
120 bp 1	130	0	1882	1251	959	14.5	9.7	7.3
120 bp 2	141	0	1432	1107	926	10.3	7.9	6.6
120 bp 3	151	0	1715	1287	1040	11.4	8.5	6.9
mean	140.7	0	1676.3	1215	975	12.1	8.7	6.9
SD	10.5	0	227.5	95.2	58.7	2.2	0.9	0.4

**Table 10:** Frequency and quality of 5S rRNA homologies in the *E. coli* K12 genome. See text for definitions of “identity”, “similarity”, etc.

5S rRNA	Count Data Total, Sum 6 Reading Frames		Sums across All Matches			Means across All Matches		
Sample	N^o^. Matches	N^o^. Perfect Identities	Total Length of Matches	Total Number of Similarities	Total Number of Identities	Mean Match Length	Mean Similarity Length	Mean Identity Length
5S rRNA	480	158	5696	4580	4136	11.5	9.5	8.6

**Table 11:** 16S rRNA protein control sequences utilized to test the frequency and quality of homologous sequences in the *E. coli* K12 genome. Sample numbers identify mRNA of control proteins of the *E. coli* K12 genome accessed from the U. C. Santa Cruz microbial genome list: http://microbes.ucsc.edu/lists/eschColi_K12/refSeq-list.html. See text for definitions of “identities”, “similarities”, etc.

Protein 16S Control	Count Data, across All Six Reading Frames		Sums across All Matches			Means across All Matches		
Sample	N^o^. Matches	N^o^. Perfect Identities	Total Length of Matches	Total Number of Similarities	Total Number of Identities	Mean Match Length	Mean Similarity Length	Mean Identity Length
b0135	389	1	10,999	7041	5647	23.8	18.1	14.5
b0070	414	1	9725	3923	5378	27.2	14.8	10.8
b0376	625	1	15,008	8069	6029	24.0	12.9	9.6
b0014	289	2	10,260	7043	5852	35.5	24.4	20.2
b0091	247	1	7924	5385	4649	32.1	21.8	18.8
mean	392.8	0.8	10,783.2	6292.2	5511	28.5	18.4	14.8
SD	147.0	0.8	2620.3	1636.9	539.4	5.2	4.8	4.7

**Table 12:** 16S rRNA random control sequences utilized to test the frequency and quality of similar sequences in the *E. coli* K12 genome. See text for definitions of “identities”, “similarities”, etc.

Random Sequence6s Control	Count Data, across All Six Reading Frames		Sums across All Matches			Means across All Matches		
Sample	N^o^. Matches	N^o^. Perfect Identities	Total Length of Matches	Total Number of Similarities	Total Number of Identities	Mean Match Length	Mean Similarity Length	Mean Identity Length
1500 bp 1	129	0	2668	1658	1347	20.7	12.9	10.4
1500 bp 2	61	0	1816	998	704	29.8	16.4	11.5
1500 bp 3	70	0	2100	1124	910	30	16.1	13
1500 bp 4	82	0	2273	1269	889	27.7	10.8	14.5
1500 bp 5	68	0	2090	1113	792	30.7	16.4	11.7
mean	82	0	2189.4	1232.4	928.4	27.8	15.4	11.5
SD	27.3	0	313.6	256.6	248	4.1	1.5	1.0

**Table 13:** Frequency and quality of 16S rRNA homologies in the *E. coli* K12 genome. See text for definitions of “identity”, “similarity”, etc.

16S rRNA	Count Data, across All Reading Frames		Sums across All Matches			Means across All Matches		
Sample	N^o^. Matches	N^o^. Perfect Identities	Total Length of Matches	Total Number of Similarities	Total Number of Identities	Mean Match Length	Mean Similarity Length	Mean Identity Length
16S rRNA	651	49	36,488	28,126	25,848	56.1	43.2	39.7

**Table 14:** 23S rRNA protein control sequences utilized to test the frequency and quality of homologous sequences in the *E. coli* K12 genome. Sample numbers identify mRNA of control proteins of the *E. coli* K12 genome accessed from the U. C. Santa Cruz microbial genome list: http://microbes.ucsc.edu/lists/eschColi_K12/refSeq-list.html. See text for definitions of “identities”, “similarities”, etc.

Protein 23S Control	Count Data, across All Six Reading Frames		Sums across All Matches			Means across All Matches		
Sample	N^o^. Matches	N^o^. Perfect Identities	Total Length of Matches	Total Number of Similarities	Total Number of Identities	Mean Match Length	Mean Similarity Length	Mean Identity Length
b0397	374	1	12,537	8947	7894	39	24	21.1
b0993	404	1	14,283	9795	8257	35.3	24.2	20.4
mean	389	1	13,405	9371	8075.5	37.2	24.1	20.8
SD	21.1	0	1234.6	599.6	256.7	2.6	0.1	0.5

**Table 15:** 23S rRNA random control sequences utilized to test the frequency and quality of similar sequences in the *E. coli* K12 genome. See text for definitions of “identities”, “similarities”, etc.

Random Sequence23S Control	Count Data, across All Six Reading Frames		Sums across All Matches			Means across All Matches		
Sample	N^o^. Matches	N^o^. Perfect Identities	Total Length of Matches	Total Number of Similarities	Total Number of Identities	Mean Match Length	Mean Similarity Length	Mean Identity Length
2900 bp 1	130	0	1882	1251	959	14.5	9.7	7.4
2900 bp 2	141	0	1432	1107	926	10.3	7.9	6.6
2900 bp 3	151	0	1715	1287	1040	11.3	8.5	6.9
2900 bp 4	117	0	1192	934	761	10.2	8.0	6.5
2900 bp 5	120	0	1235	958	805	10.3	8.0	6.7
mean	131.8	0	1491.2	1107.4	898.2	11.3	8.4	6.8
SD	14.3	0	300.6	162.2	114.1	1.8	0.7	0.4

**Table 16:** Frequency and quality of 23S rRNA homologies in the *E. coli* K12 genome. See text for definitions of “identity”, “similarity”, etc.

23S rRNA	Count Data, across All Reading Frames		Sums across All Matches			Means across All Matches		
Sample	N^o^. Matches	N^o^. Perfect Identities	Total Length of Matches	Total Number of Similarities	Total Number of Identities	Mean Match Length	Mean Similarity Length	Mean Identity Length
23S rRNA	195	18	41,096	38,529	38,119	210.7	197.6	192.9
